# Causal relationship between mitochondrial-associated proteins and cerebral aneurysms: a Mendelian randomization study

**DOI:** 10.3389/fneur.2024.1405086

**Published:** 2024-07-17

**Authors:** Shuai Wang, Jiajun Wang, Zihui Niu, Kang Zhang, Tao Yang, Shiqiang Hou, Ning Lin

**Affiliations:** Department of Neurosurgery, The Affiliated Chuzhou Hospital of Anhui Medical University, The First People's Hospital of Chuzhou, Chuzhou, Anhui, China

**Keywords:** cerebral aneurysms, mitochondrial-associated proteins, Mendelian randomization, GWAS data, causal association

## Abstract

**Background:**

Cerebral aneurysm is a high-risk cerebrovascular disease with a poor prognosis, potentially linked to multiple factors. This study aims to explore the association between mitochondrial-associated proteins and the risk of cerebral aneurysms using Mendelian randomization (MR) methods.

**Methods:**

We used GWAS summary statistics from the IEU Open GWAS project for mitochondrial-associated proteins and from the Finnish database for cerebral aneurysms (uIA, aSAH). The association between mitochondrial-associated exposures and cerebral aneurysms was evaluated using MR-Egger, weighted mode, IVW, simple mode and weighted median methods. Reverse MR assessed reverse causal relationship, while sensitivity analyses examined heterogeneity and pleiotropy in the instrumental variables. Significant causal relationship with cerebral aneurysms were confirmed using FDR correction.

**Results:**

Through MR analysis, we identified six mitochondrial proteins associated with an increased risk of aSAH: AIF1 (OR: 1.394, 95% CI: 1.109–1.752, *p* = 0.0044), CCDC90B (OR: 1.318, 95% CI: 1.132–1.535, *p* = 0.0004), TIM14 (OR: 1.272, 95% CI: 1.041–1.553, *p* = 0.0186), NAGS (OR: 1.219, 95% CI: 1.008–1.475, *p* = 0.041), tRNA PusA (OR: 1.311, 95% CI: 1.096–1.569, *p* = 0.003), and MRM3 (OR: 1.097, 95% CI: 1.016–1.185, *p* = 0.0175). Among these, CCDC90B, tRNA PusA, and AIF1 demonstrated a significant causal relationship with an increased risk of aSAH (FDR *q* < 0.1). Three mitochondrial proteins were associated with an increased risk of uIA: CCDC90B (OR: 1.309, 95% CI: 1.05–1.632, *p* = 0.0165), tRNA PusA (OR: 1.306, 95% CI: 1.007–1.694, *p* = 0.0438), and MRM3 (OR: 1.13, 95% CI: 1.012–1.263, *p* = 0.0303). In the reverse MR study, only one mitochondrial protein, TIM14 (OR: 1.087, 95% CI: 1.004–1.177, *p* = 0.04), showed a causal relationship with aSAH. Sensitivity analysis did not reveal heterogeneity or pleiotropy. The results suggest that CCDC90B, tRNA PusA, and MRM3 may be common risk factors for cerebral aneurysms (ruptured and unruptured), while AIF1 and NAGS are specifically associated with an increased risk of aSAH, unrelated to uIA. TIM14 may interact with aSAH.

**Conclusion:**

Our findings confirm a causal relationship between mitochondrial-associated proteins and cerebral aneurysms, offering new insights for future research into the pathogenesis and treatment of this condition.

## Introduction

The incidence of cerebral aneurysms is about 3–5%, characterized by degenerative changes in the arterial wall and destruction of elastic and collagen fibers. This leads to abnormal local dilatation of the vessel wall, with rupture being the most serious complication (1). After rupture, blood is introduced into the subarachnoid space under high pressure, resulting in aneurysmal subarachnoid hemorrhage (aSAH) (2). Although aSAH accounts for only 5% of all strokes, its mortality rate is as high as 40% (3). Hemorrhagic aneurysms can be treated with surgical clipping or endovascular coiling. However, many patients still experience poor clinical outcomes due to various complications (4). The etiology and treatment of cerebral aneurysms warrant further in-depth study.

The relationship between mitochondria and cerebral aneurysms has garnered significant attention in recent years. Research suggests that mitochondrial DNA (mtDNA) could impact complications and clinical outcomes after aSAH (5). Mitochondria are important regulators of cellular apoptosis (6). Abnormal mitochondrial ultrastructure, defects in the electron transport chain, increased reactive oxygen species (ROS), oxidative stress, and unique dynamic characteristics play crucial roles in regulating the process of cellular apoptosis (7). Notably, mitochondria-induced necrotic apoptosis is implicated in the formation of cerebral aneurysms (8). and mitochondrial apoptosis triggered by respiratory chain dysfunction serves as a pathological mechanism of cerebral aneurysms (9). Mitochondrial dysfunction is the primary culprit for neuronal damage following early cerebral ischemic events (10). During the acute phase following SAH, mitochondrial calcium overload can lead to a substantial generation of ROS, resulting in cell death. Inhibiting the mitochondrial calcium uniporter protein (MCU) or preventing calcium accumulation significantly mitigates oxidative damage and reduces neuronal cell death (11). Furthermore, mitochondrial dysfunction stemming from impaired mitochondrial function is a significant contributor to microvascular and macrovascular diseases, including those affecting cerebral arteries, carotid arteries, and the aorta (12, 13). However, the causal relationship between mitochondrial function and cerebral aneurysms has yet to be definitively established.

Mendelian randomization (MR) is a method used to evaluate causality by leveraging genetic variation. Because genetic variation is determined at fertilization and is typically independent of environmental factors, MR can offer causal inferences that closely resemble random assignments, thereby minimizing the influence of confounding variables (14). In this study, we employed MR to investigate the potential causal relationship between mitochondrial exposure and cerebral aneurysms.

## Materials and methods

### Data source

The Single Nucleotide Polymorphisms (SNPs) associated with mitochondria were obtained from IEU Open GWAS project. The project provides publicly available summary statistics data on 66 mitochondrial-associated proteins from 3,301 European ancestry healthy blood donors in the INTERVAL study. All participants provided informed consent under national research ethics, and underwent extensive surveys on demographic characteristics, including age, gender, anthropometric measurements such as height and weight, lifestyle factors (e.g., alcohol consumption and smoking), and dietary habits. Additionally, individuals with major diseases (e.g., myocardial infarction, stroke, cancer) or recent illnesses or infections were excluded based on blood donation criteria (15). To minimize potential biases due to sample overlap, we chose genetic association data related to cerebral aneurysms (unruptured intracranial aneurysm (uIA) and aSAH) from the Finnish database. This database can be accessed online through the IEU Open GWAS project website.^1^ The dataset for uIA included 992 cases and 203,068 controls of European ancestry, with the accession number finn-b-I9_ANEURYSM. Similarly, the dataset for aSAH comprised 2,127 cases and 203,068 controls of European ancestry, with the accession number finn-b-I9_SAHANEUR. Although detailed demographic variables (such as age and gender) of the samples are not directly provided in the database, we will conduct analyses based on summary statistics data related to genetic variations and phenotype associations, including *p*-values, effect sizes, and standard errors provided by the database.

### Instrumental variables selection

The selection of instrumental variables (IVs) was based on significance levels below *p* < 5 ×10^−6^, utilizing a clumping window size of 10,000 kb and a threshold of r^2^ < 0.001 to minimize linkage disequilibrium (LD). To assess the statistical strength of the IVs, the formula F = (R^2^(n–k–1)) / (k(1–R^2^)) was employed, where n signifies the count of samples that have been exposed to the variables under investigation, k represents the number of IVs employed in the study, and R^2^ value quantifies the proportion of variance in exposure that can be attributed to the IVs, indicating the extent to which these IVs explain the observed variation in the exposure of interest (16). A weakly relevant instrumental variable was considered when *F* < 10 (17). Notably, all SNPs utilized displayed *F* values exceeding 10, indicative of a robust correlation among the IVs employed.

### Statistical analysis

We conducted an investigation into the relationship between mitochondrial-associated exposures and cerebral aneurysms utilizing five methods: MR-Egger, weighted mode, Inverse Variance Weighting (IVW), simple mode and weighted median. Among these, IVW is the most widely used MR method today (18). Assuming all IVs are valid, IVW is the most efficient method. It estimates the causal effect of exposure on the outcome by performing a weighted average of the effect estimates from each IV, with more precise estimates (those with smaller standard errors) contributing more to the final result, thus making the estimates more accurate and reliable (19). Therefore, we present IVW analysis as the primary result. To ensure the rigor and completeness of the study, we included four additional methods to complement the IVW results.MR-Egger regression is specifically designed to detect and adjust for horizontal pleiotropy in causal effect analyses, providing consistent estimates of causal effects under the weaker InSIDE assumption, even in the presence of invalid instruments (20). The weighted mode method aims to provide robust causal effect estimates by identifying and using the most common weighted values among the IVs, reducing the impact of outliers and bias. The simple mode method assigns equal weight to all instrumental variables and is straightforward in its calculation, not involving complex statistical models or weighting processes, but it may be influenced by outliers or low-precision estimates. The weighted median method yields more effective results when more than 50% of the instrumental variables are valid (21).

Furthermore, sensitivity testing was performed, including the examination of horizontal pleiotropy using the MR-Egger intercept test and the MR-PRESSO test. A statistically significant *p* value less than 0.05 indicated evidence of horizontal pleiotropy among the included SNPs (22). Heterogeneity was assessed using Cochran’s Q test, where *p*-values less than 0.05 indicate the presence of heterogeneity (23). Crucially, leave-one-out sensitivity analyses were performed to underscore the robustness and reliability of our findings. These analyses revealed that excluding any individual SNP did not yield a significant impact on the overall results, highlighting the consistency of our findings. To assess the reverse causal effect between mitochondrial-associated proteins and cerebral aneurysms, we examined cerebral aneurysms as the “exposure” and mitochondrial-associated proteins as the “outcome.” SNPs significantly associated with aSAH and uIA (*p* < 5 × 10^−6^) were selected as IVs. In the end, we utilized false discovery rate (FDR) correction to obtain adjusted *p*-values, denoted as q (24). We define that when *q* < 0.1, it indicates a significant causal relationship between exposure and outcome; whereas when *p* < 0.05 and *q* ≥ 0.1, it suggests an indicative causal effect (25). The study followed the STROBE-MR guidelines, ensuring rigorous adherence to standardized reporting and methodology (26), and two-sample MR analysis was conducted utilizing the “TwoSampleMR” package in R version 4.3.1.

## Results

### Causal effect between mitochondrial-associated proteins and aneurysms

**Figure 1:**
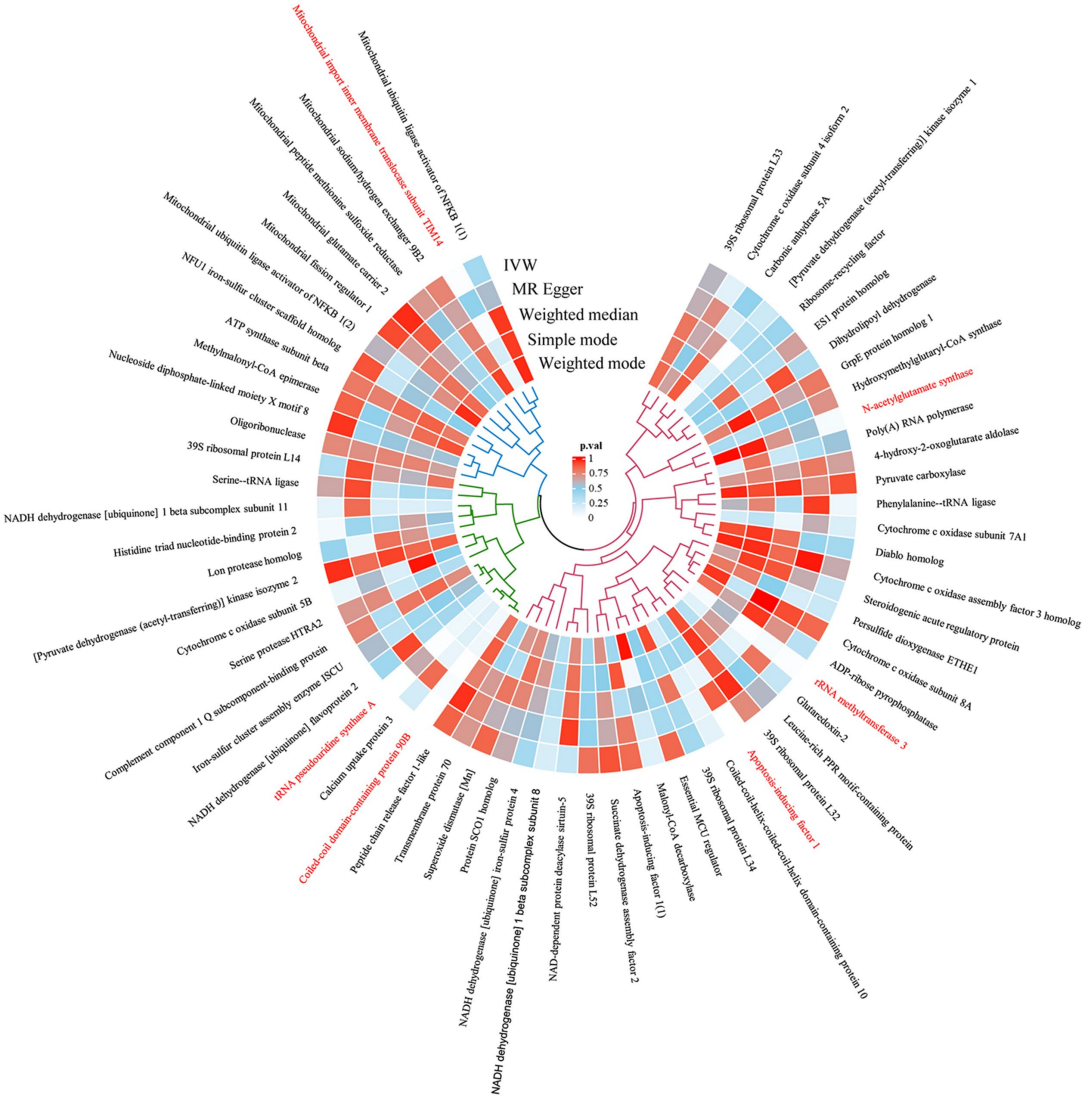
The circular heatmap depicts the results of utilizing MR method to analyze the causal relationship between 66 mitochondrial-associated proteins and aSAH.

**Figure 2:**
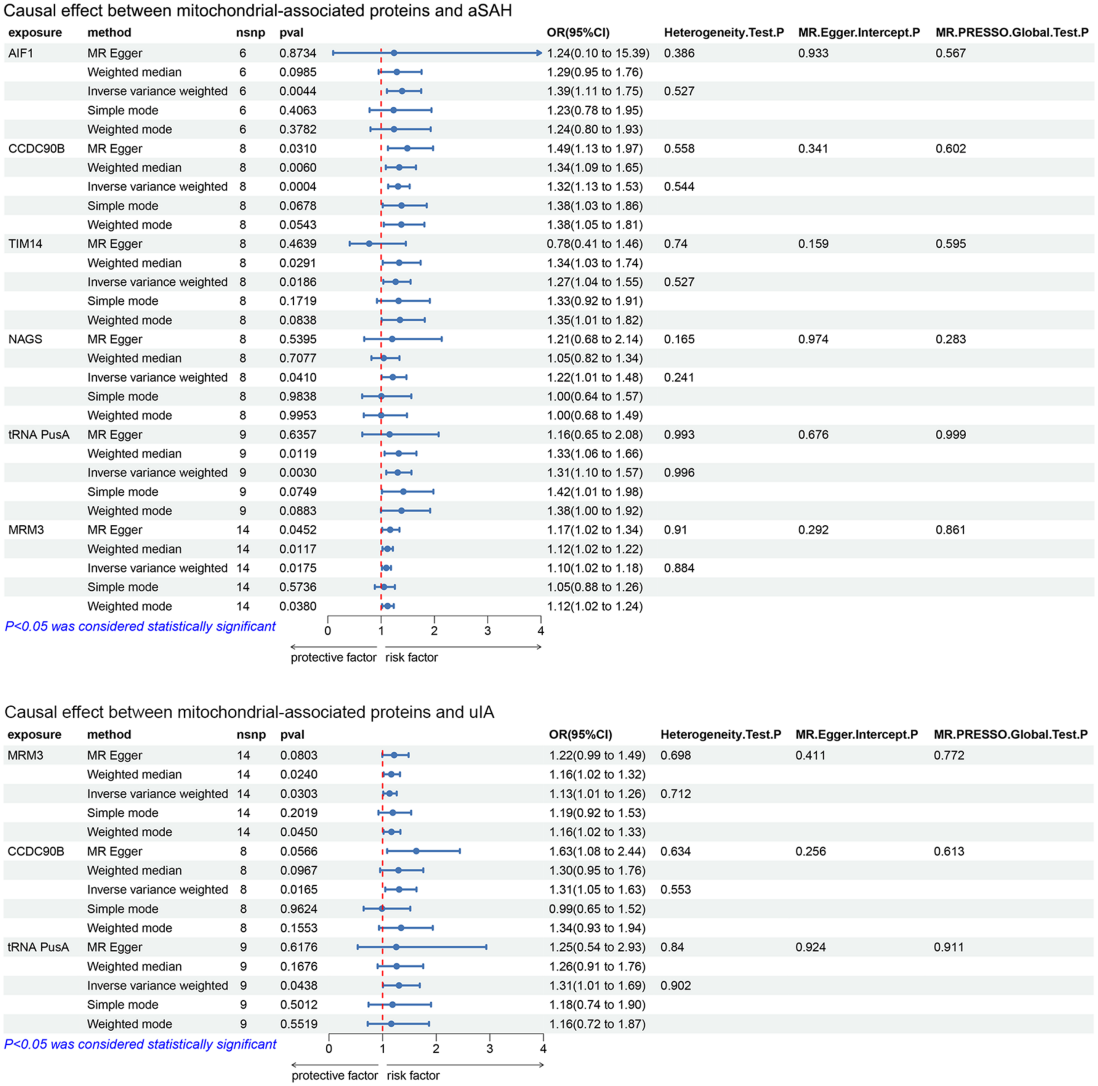
Forest plot results of the analysis of the relationship between mitochondrial-associated proteins and cerebral aneurysms (aSAH, uIA) using MR method (IVW *p* < 0.05).

The MR analysis investigated the relationship between mitochondrial-associated proteins and cerebral aneurysms using five methods: MR-Egger, weighted mode, IVW, simple mode and weighted median, with IVW as the primary analytical method. According to the IVW method, MR analysis showed that in aSAH, AIF1 (OR: 1.394, 95%CI:1.109–1.752, *p* = 0.0044), CCDC90B (OR: 1.318, 95%CI: 1.132–1.535, *p* = 0.0004), TIM14 (OR: 1.272, 95%CI: 1.041–1.553, *p* = 0.0186), NAGS (OR:1.219, 95%CI:1.008–1.475,*p* = 0.041), tRNA PusA (OR:1.311, 95%CI:1.096–1.569, *p* = 0.003), MRM3 (OR:1.097, 95%CI:1.016–1.185, *p* = 0.0175), the genetic prediction of these six mitochondrial-associated proteins suggests causal association with aSAH, this implies that variations in these proteins may indeed influence the risk or development of aSAH (Figures 1, 2). In uIA, the genetic prediction of CCDC90B (OR:1.309, 95%CI: 1.05–1.632, *p* = 0.0165), tRNA PusA (OR: 1.306, 95%CI: 1.007–1.694, *p* = 0.0438) and MRM3 (OR: 1.13, 95%CI: 1.012–1.263, *p* = 0.0303) was associated with an increased risk of uIA.

We conducted Cochran’s Q test, revealing that none of the mitochondrial-associated protein variables exhibited significance. This indicates the absence of heterogeneity among the IVs. Subsequently, multiple validity tests were performed using MR-PRESSO, which did not identify any outliers in the data. In the MR-Egger intercept test, we investigated whether the remaining IVs still displayed pleiotropy. The analysis of intercepts did not yield statistically significant results, suggesting a lack of meaningful associations and indicating the absence of horizontal pleiotropy. This implies that any observed relationships between variables are less likely to be influenced by unintended genetic effects, enhancing the validity of the findings (Figure 2). These sensitivity analyses provide further support for the reliability and robustness of our findings regarding the causal relationships between mitochondrial-associated proteins and cerebral aneurysms.

To correct for potential biases caused by multiple comparisons, we applied FDR correction. The results revealed that in aSAH, CCDC90B had a q-value of 0.024, AIF1 had a q-value of 0.096, and tRNA PusA had a q-value of 0.099. This indicates significant causal effects of CCDC90B, AIF1, and RNA PusA in aSAH, while results with q-values greater than or equal to 0.1 suggest indicative causal effects.

### Reverse causal effect between mitochondrial-associated proteins and cerebral aneurysms

**Figure 3:**
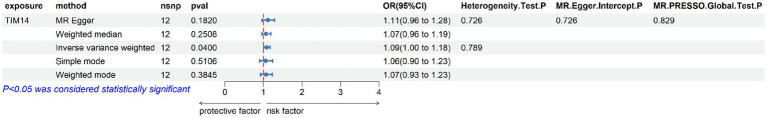
Forest plot of the reverse causal relationship between mitochondrial-associated proteins and aSAH.

**Figure 4:**
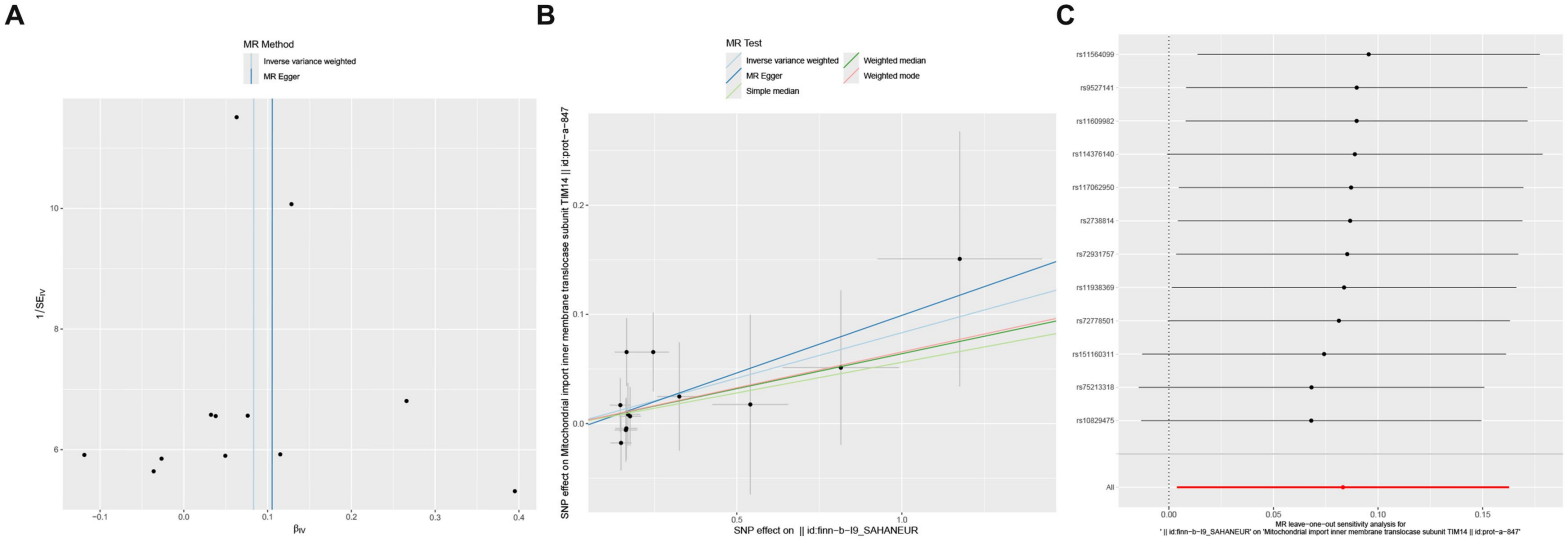
Reverse causal effect between mitochondrial-associated proteins and aSAH, including scatter plot **(A)**, funnel plot **(B)** and leave-one-out result **(C)**.

Reverse MR analysis was conducted on the six mitochondrial-associated proteins previously identified as causally associated with cerebral aneurysms in the forward MR analysis, to investigate the reverse causal relationship between mitochondrial-associated proteins and cerebral aneurysms. The reverse MR analysis revealed that in cases of aSAH, the occurrence of aSAH was positively and significantly associated with the presence of TIM14 (OR: 1.087, 95% CI: 1.004–1.177, *p* = 0.04). In sensitivity analysis, both Cochran’s Q test and MR-Egger intercept test and MR-PRESSO global tests did not reveal significant heterogeneity or horizontal pleiotropy (Figure 3). Similarly, no notable biases or differences were observed in the scatter plot, funnel plot and leave-one-out analysis (Figure 4). No inverse causal relationship was observed between the remaining mitochondrial-associated proteins and aSAH or uIA.

## Discussion

This study employed MR analysis to examine the causal relationship between 66 mitochondrial-associated proteins and aSAH and uIA. Utilizing publicly available data from IEU Open GWAS project and the Finnish database, and incorporating results adjusted for FDR, we identified six mitochondria proteins (AIF1, CCDC90B, TIM14, NAGS, tRNA PusA, MRM3) associated with increased risk of aSAH. Among these, CCDC90B, tRNA PusA, and AIF1 demonstrated a significant causal relationship with an increased risk of aSAH (*q* < 0.1). Additionally, three proteins (CCDC90B, tRNA PusA, MRM3) were associated with increased risk of uIA. In the reverse analysis, only one mitochondria protein (TIM14) exhibited a causal relationship with aSAH. Overall, our findings suggest that CCDC90B, tRNA PusA, and MRM3 may be common risk factors for cerebral aneurysms (ruptured and unruptured), while AIF1 and NAGS are specifically associated with an increased risk of aSAH, unrelated to uIA. TIM14 may be involved in interactions related to aSAH.

The study on the relationship between mitochondrial-associated proteins and cerebral aneurysms holds significant clinical implications. Apoptosis-inducing factor 1(AIF1), initially discovered as a flavoprotein located in the mitochondrial intermembrane space, was first identified as a cell apoptosis-inducing factor (27, 28). After translocation from mitochondria to the nucleus, AIF1 can induce DNA fragmentation and cell apoptosis through a caspase-independent mechanism, leading to apoptosis within the cell or affecting cell survival by modulating mitochondrial function (29, 30), and the mitochondria-necrotic apoptosis axis is one of the pathogenic mechanisms of cerebral aneurysms (8). Numerous experimental studies have demonstrated that AIF is the primary cause of ischemic and traumatic neuronal death. Research involving animal models has revealed that mice with the AIF1 gene suppressed exhibit a decrease in the size of brain lesions after temporary localized cerebral ischemia when compared to their genetically unmodified counterparts. Additionally, these mice experienced a reduction in neuronal death in the ischemic region following transient cerebral artery occlusion. However, AIF1 protein levels more than double after hypoxia-ischemia (HI), leading to more severe brain damage (27, 29). In line with our research findings, AIF1 may be a significant factor contributing to cerebral aneurysms, particularly aSAH. N-acetylglutamate synthase (NAGS), a potential regulatory urea cycle protein present in the mitochondrial matrix, is involved in the urea cycle, which is a metabolic pathway within organisms, primarily responsible for converting toxic ammonia into urea. Dysregulation of this cycle can lead to abnormal brain function (31, 32). NAGS may influence the occurrence and development of cerebral aneurysms through the urea cycle metabolic pathway, and all of this needs further mechanistic research for validation in the future.

tRNA pseudouridine synthase A (tRNA PusA) belongs to the RNA modification enzyme family. Its function is to catalyze the conversion of uridine nucleotides in tRNA molecules to pseudouridine nucleotides. Abnormal tRNA PusA modification in mitochondria can lead to mitochondrial dysfunction, resulting in severe pathological consequences, including mitochondrial myopathy, encephalopathy, mitochondrial encephalomyopathy with lactic acidosis and stroke-like episodes (MELAS), among other mitochondrial diseases (33–35), highlighting its critical biological function in brain mitochondria (36). A clinical study revealed a correlation between mitochondrial diseases, particularly MELAS (37), and cerebral aneurysms. Mitochondrial diseases lead to increased oxidative and nitrosative stress in the vascular wall, which may be a significant factor in the development of cerebral aneurysms (12, 13). This suggests the important role of aberrant tRNA PusA expression in the progression of cerebral aneurysms. Coiled-coil domain-containing protein 90B (CCDC90B) is a protein containing a coiled-coil domain involved in maintaining cellular structure, signal transduction, and gene expression regulation (38). Given the limited research on CCDC90B, further studies are needed to elucidate its mechanism of action in cerebral aneurysms. rRNA methyltransferase 3 (MRM3) is an important mitochondrial methyltransferase involved in ribosomal RNA modification and participates in ribosome assembly, affecting ribosomal translation activity and protein synthesis rates. It is a crucial factor influencing protein synthesis and OXPHOS function in mitochondria (39). Dysfunction of OXPHOS function is associated with apoptosis (40), the mechanism may involve MRM3 influencing cell apoptosis through mitochondrial oxidative phosphorylation, thereby affecting the development of cerebral aneurysms. Mitochondrial import inner membrane translocase subunit TIM14 (TIM14) is a protein located on the mitochondrial membrane and is one of the components of the mitochondrial protein translocation complex, closely associated with mitochondrial protein transport and folding processes (41). Research results indicate a bidirectional causal relationship between TIM14 and cerebral aneurysms, suggesting its interaction with cerebral aneurysms; however, further investigation is needed to elucidate its underlying mechanisms.

The causal relationship between mitochondrial-associated proteins and cerebral aneurysms has been identified through MR analysis, laying the foundation for in-depth research into the association between mitochondria and cerebral aneurysms. This discovery opens up new directions and insights for future studies on the etiology and mechanisms of cerebral aneurysms. It may facilitate the discovery of new therapeutic targets and the development of more effective preventive strategies, providing a breakthrough for the prevention and treatment of cerebral aneurysms.

However, we must consider the limitations of this study. The dataset utilized in this study primarily consists of individuals of European ancestry, which may restrict the broader applicability of the findings due to the absence of more diverse demographic information such as gender and age. Consequently, the generalizability of the results to wider populations is limited, and there are constraints on the potential for further analysis. Additionally, we did not include multivariable MR analysis. Considering the limitations in sample size and data completeness, the statistical power for multivariable MR analysis might be insufficient. Future research should aim to increase sample size and improve data completeness, incorporating multivariable MR analysis to more comprehensively understand the combined effects of these proteins in the formation of cerebral aneurysms. Finally, while mitochondria play a crucial role in the development of cerebral aneurysms, our study revealed causal relationships between mitochondrial-associated proteins and cerebral aneurysms. Further research is imperative to deepen our understanding of the intricate mechanisms underlying cerebral aneurysms.

## Conclusion

Through MR analysis, we have established a causal relationship between mitochondrial-associated proteins and cerebral aneurysms. This finding lays the groundwork for further exploration into the potential mechanisms linking mitochondria to cerebral aneurysms. It provides new directions and insights for future research on the etiology and mechanisms of cerebral aneurysms, and may offer new perspectives for future prevention and treatment strategies.

## Data availability statement

The original contributions presented in the study are included in the article/Supplementary material, further inquiries can be directed to the corresponding author.

## Ethics statement

Ethical review and approval were not required for the study on human participants in accordance with the local legislation and institutional requirements. Written informed consent from the patients/participants or patients/participants' legal guardian/next of kin was not required to participate in this study in accordance with the national legislation and the institutional requirements.

## Author contributions

SW: Writing – original draft. JW: Writing – review & editing. ZN: Writing – original draft. KZ: Writing – review & editing. TY: Writing – review & editing. SH: Writing – original draft. NL: Writing – review & editing.

## Footnotes

^1^ https://gwas.mrcieu.ac.uk/

## References

[1] Chalouhi N Hoh BL Hasan D. Review of cerebral aneurysm formation, growth, and rupture. Stroke. (2013) 44:3613–22. doi: 10.1161/STROKEAHA.113.002390 24130141

[2] Macdonald RL. Delayed neurological deterioration after subarachnoid haemorrhage. Nat Rev Neurol. (2014) 10:44–58. doi: 10.1038/nrneurol.2013.246 24323051

[3] Macdonald RL Schweizer TA. Spontaneous subarachnoid haemorrhage. Lancet. (2017) 389:655–66. doi: 10.1016/S0140-6736(16)30668-7 27637674

[4] Chaudhry SR Hafez A Rezai Jahromi B Kinfe TM Lamprecht A Niemela M. Role of damage associated molecular pattern molecules (DAMPs) in aneurysmal subarachnoid hemorrhage (aSAH). Int J Mol Sci. (2018) 19. doi: 10.3390/ijms19072035, PMID: 30011792 PMC6073937

[5] Chaudhry SR Frede S Seifert G Kinfe TM Niemela M Lamprecht A. Temporal profile of serum mitochondrial DNA (mt DNA) in patients with aneurysmal subarachnoid hemorrhage (aSAH). Mitochondrion. (2019) 47:218–26. doi: 10.1016/j.mito.2018.12.001, PMID: 30529453

[6] Bock FJ Tait SWG. Mitochondria as multifaceted regulators of cell death. Nat Rev Mol Cell Biol. (2020) 21:85–100. doi: 10.1038/s41580-019-0173-8 31636403

[7] Kaur MM Sharma DS. Mitochondrial repair as potential pharmacological target in cerebral ischemia. Mitochondrion. (2022) 63:23–31. doi: 10.1016/j.mito.2022.01.001, PMID: 34999014

[8] Chen B Xie K Zhang J Yang L Zhou H Zhang L. Comprehensive analysis of mitochondrial dysfunction and necroptosis in intracranial aneurysms from the perspective of predictive, preventative, and personalized medicine. Apoptosis. (2023) 28:1452–68. doi: 10.1007/s10495-023-01865-x, PMID: 37410216 PMC10425526

[9] Finsterer J. Familial intracranial aneurysm requires not only whole-exome sequencing, but also mitochondrial DNA sequencing. Korean J Radiol. (2022) 23:566–7. doi: 10.3348/kjr.2022.0029, PMID: 35506531 PMC9081687

[10] Gong Z Pan J Shen Q Li M Peng Y. Mitochondrial dysfunction induces NLRP3 inflammasome activation during cerebral ischemia/reperfusion injury. J Neuroinflammation. (2018) 15:242. doi: 10.1186/s12974-018-1282-6, PMID: 30153825 PMC6114292

[11] Yan H Zhang D Hao S Li K Hang CH. Role of mitochondrial calcium uniporter in early brain injury after experimental subarachnoid hemorrhage. Mol Neurobiol. (2015) 52:1637–47. doi: 10.1007/s12035-014-8942-z, PMID: 25370932

[12] Finsterer J Mahjoub SZ. Primary mitochondrial arteriopathy. Nutr Metab Cardiovasc Dis. (2012) 22:393–9. doi: 10.1016/j.numecd.2012.01.002, PMID: 22520486

[13] Vattemi G Mechref Y Marini M Tonin P Minuz P Grigoli L. Increased protein nitration in mitochondrial diseases: evidence for vessel wall involvement. Mol Cell Proteomics. (2011) 10:M110.002964. doi: 10.1074/mcp.M110.002964, PMID: 21156839 PMC3069340

[14] Sekula P Del Greco MF Pattaro C Kottgen A. Mendelian randomization as an approach to assess causality using observational data. J Am Soc Nephrol. (2016) 27:3253–65. doi: 10.1681/ASN.2016010098, PMID: 27486138 PMC5084898

[15] Sun BB Maranville JC Peters JE Stacey D Staley JR Blackshaw J. Genomic atlas of the human plasma proteome. Nature. (2018) 558:73–9. doi: 10.1038/s41586-018-0175-2, PMID: 29875488 PMC6697541

[16] Palmer TM Lawlor DA Harbord RM Sheehan NA Tobias JH Timpson NJ. Using multiple genetic variants as instrumental variables for modifiable risk factors. Stat Methods Med Res. (2012) 21:223–42. doi: 10.1177/0962280210394459, PMID: 21216802 PMC3917707

[17] Burgess S Small DS Thompson SG. A review of instrumental variable estimators for Mendelian randomization. Stat Methods Med Res. (2017) 26:2333–55. doi: 10.1177/0962280215597579, PMID: 26282889 PMC5642006

[18] Lin Z Deng Y Pan W. Combining the strengths of inverse-variance weighting and Egger regression in Mendelian randomization using a mixture of regressions model. PLoS Genet. (2021) 17:e1009922. doi: 10.1371/journal.pgen.1009922, PMID: 34793444 PMC8639093

[19] Dudbridge F. Polygenic Mendelian randomization. Cold Spring Harb Perspect Med. (2021) 11:2. doi: 10.1101/cshperspect.a039586 PMC7849343 32229610

[20] Burgess S Thompson SG. Interpreting findings from Mendelian randomization using the MR-Egger method. Eur J Epidemiol. (2017) 32:377–89. doi: 10.1007/s10654-017-0255-x, PMID: 28527048 PMC5506233

[21] Bowden J Davey Smith G Haycock PC Burgess S. Consistent estimation in Mendelian randomization with some invalid instruments using a weighted median estimator. Genet Epidemiol. (2016) 40:304–14. doi: 10.1002/gepi.21965, PMID: 27061298 PMC4849733

[22] Bowden J Del Greco MF Minelli C Davey Smith G Sheehan NA Thompson JR. Assessing the suitability of summary data for two-sample Mendelian randomization analyses using MR-Egger regression: the role of the I2 statistic. Int J Epidemiol. (2016) 45:1961–74. doi: 10.1093/ije/dyw220, PMID: 27616674 PMC5446088

[23] Burgess S Bowden J Fall T Ingelsson E Thompson SG. Sensitivity analyses for robust causal inference from Mendelian randomization analyses with multiple genetic variants. Epidemiology. (2017) 28:30–42. doi: 10.1097/EDE.0000000000000559, PMID: 27749700 PMC5133381

[24] Storey JD Tibshirani R. Statistical significance for genomewide studies. Proc Natl Acad Sci USA. (2003) 100:9440–5. doi: 10.1073/pnas.1530509100, PMID: 12883005 PMC170937

[25] Chen H Ye B Su W Song Y Sun PL Zhou X. The causal role of gut microbiota in susceptibility and severity of COVID-19: a bidirectional Mendelian randomization study. J Med Virol. (2023) 95:e28880. doi: 10.1002/jmv.28880, PMID: 37409643

[26] Skrivankova VW Richmond RC Woolf BAR Yarmolinsky J Davies NM Swanson SA. Strengthening the reporting of observational studies in epidemiology using Mendelian randomization: the STROBE-MR statement. JAMA. (2021) 326:1614–21. doi: 10.1001/jama.2021.18236, PMID: 34698778

[27] Li T Li K Zhang S Wang Y Xu Y Cronin SJF. Overexpression of apoptosis inducing factor aggravates hypoxic-ischemic brain injury in neonatal mice. Cell Death Dis. (2020) 11:77. doi: 10.1038/s41419-020-2280-z, PMID: 32001673 PMC6992638

[28] Sevrioukova IF. Apoptosis-inducing factor: structure, function, and redox regulation. Antioxid Redox Signal. (2011) 14:2545–79. doi: 10.1089/ars.2010.3445, PMID: 20868295 PMC3096518

[29] Culmsee C Zhu C Landshamer S Becattini B Wagner E Pellecchia M. Apoptosis-inducing factor triggered by poly (ADP-ribose) polymerase and bid mediates neuronal cell death after oxygen-glucose deprivation and focal cerebral ischemia. J Neurosci. (2005) 25:10262–72. doi: 10.1523/JNEUROSCI.2818-05.2005, PMID: 16267234 PMC6725791

[30] Cande C Cohen I Daugas E Ravagnan L Larochette N Zamzami N. Apoptosis-inducing factor (AIF): a novel caspase-independent death effector released from mitochondria. Biochimie. (2002) 84:215–22. doi: 10.1016/S0300-9084(02)01374-3, PMID: 12022952

[31] Haskins N Bhuvanendran S Anselmi C Gams A Kanholm T Kocher KM. Mitochondrial enzymes of the urea cycle cluster at the inner mitochondrial membrane. Front Physiol. (2020) 11:542950. doi: 10.3389/fphys.2020.542950 33551825 PMC7860981

[32] Natesan V Mani R Arumugam R. Clinical aspects of urea cycle dysfunction and altered brain energy metabolism on modulation of glutamate receptors and transporters in acute and chronic hyperammonemia. Biomed Pharmacother. (2016) 81:192–202. doi: 10.1016/j.biopha.2016.04.010, PMID: 27261594

[33] Gorman GS Chinnery PF DiMauro S Hirano M Koga Y McFarland R. Mitochondrial diseases. Nat Rev Dis Primers. (2016) 2:16080. doi: 10.1038/nrdp.2016.80 27775730

[34] Cui W Zhao D Jiang J Tang F Zhang C Duan C. tRNA modifications and modifying enzymes in disease, the potential therapeutic targets. Int J Biol Sci. (2023) 19:1146–62. doi: 10.7150/ijbs.80233, PMID: 36923941 PMC10008702

[35] Bykhovskaya Y Casas K Mengesha E Inbal A Fischel-Ghodsian N. Missense mutation in pseudouridine synthase 1 (PUS1) causes mitochondrial myopathy and sideroblastic anemia (MLASA). Am J Hum Genet. (2004) 74:1303–8. doi: 10.1086/421530, PMID: 15108122 PMC1182096

[36] Rintala-Dempsey AC Kothe U. Eukaryotic stand-alone pseudouridine synthases - RNA modifying enzymes and emerging regulators of gene expression? RNA Biol. (2017) 14:1185–96. doi: 10.1080/15476286.2016.1276150, PMID: 28045575 PMC5699540

[37] Zhu K Li S Chen H Wang Y Yu M Wang H. Late onset MELAS with m.3243A > G mutation and its association with aneurysm formation. Metab Brain Dis. (2017) 32:1069–72. doi: 10.1007/s11011-017-9989-0, PMID: 28321601

[38] Adlakha J Karamichali I Sangwallek J Deiss S Bar K Coles M. Characterization of MCU-binding proteins MCUR1 and CCDC90B-representatives of a protein family conserved in prokaryotes and eukaryotic organelles. Structure. (2019) 27:464–475.e6. doi: 10.1016/j.str.2018.11.004, PMID: 30612859

[39] Rorbach J Boesch P Gammage PA Nicholls TJ Pearce SF Patel D. MRM2 and MRM3 are involved in biogenesis of the large subunit of the mitochondrial ribosome. Mol Biol Cell. (2014) 25:2542–55. doi: 10.1091/mbc.e14-01-0014, PMID: 25009282 PMC4148245

[40] Schull S Gunther SD Brodesser S Seeger JM Tosetti B Wiegmann K. Cytochrome c oxidase deficiency accelerates mitochondrial apoptosis by activating ceramide synthase 6. Cell Death Dis. (2015) 6:e1691. doi: 10.1038/cddis.2015.62, PMID: 25766330 PMC4385940

[41] Mokranjac D Sichting M Neupert W Hell K. Tim14, a novel key component of the import motor of the TIM23 protein translocase of mitochondria. EMBO J. (2003) 22:4945–56. doi: 10.1093/emboj/cdg485, PMID: 14517234 PMC204468

