# Supplementary material for: Causal relationship between mitochondrial-associated proteins and cerebral aneurysms: a Mendelian randomization study
Source: Front Neurol. 2024 Jul 17;15:1405086. doi: 10.3389/fneur.2024.1405086 (PMC11290338; doi:10.3389/fneur.2024.1405086)

Causal effect between mitochondrial-associated proteins and aSAH, including scatter plots, funnel plots and leave-one-out results

AIF1

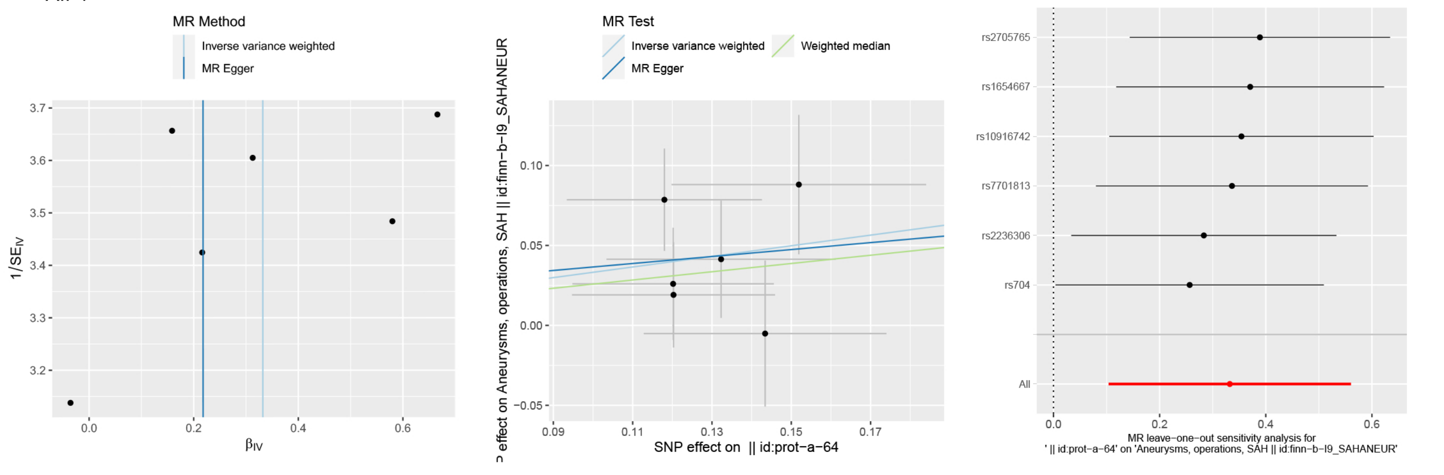

CCDC90B

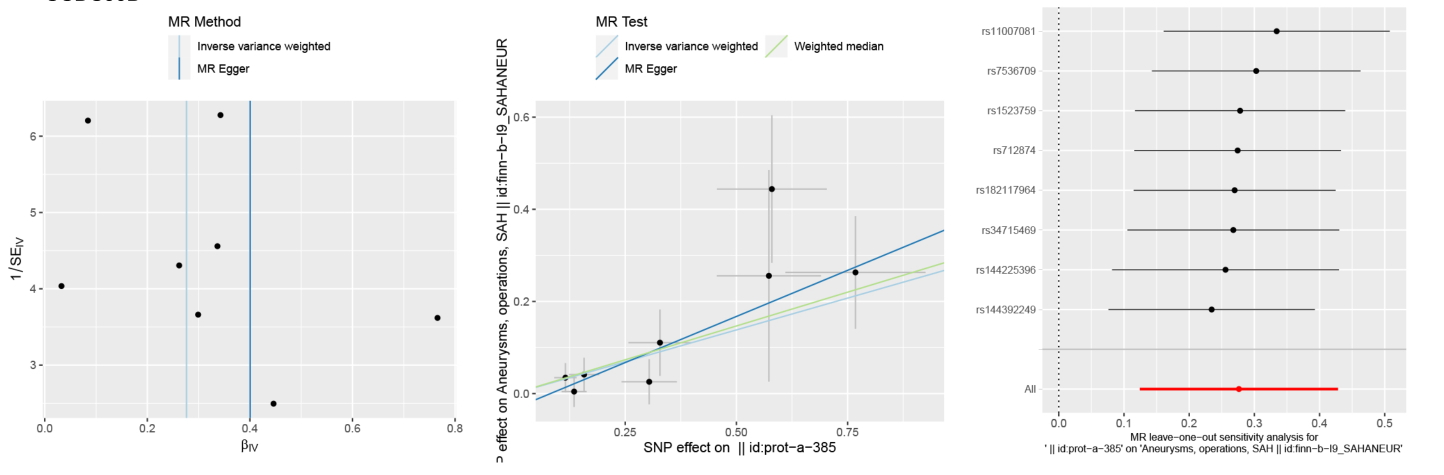

TIM14

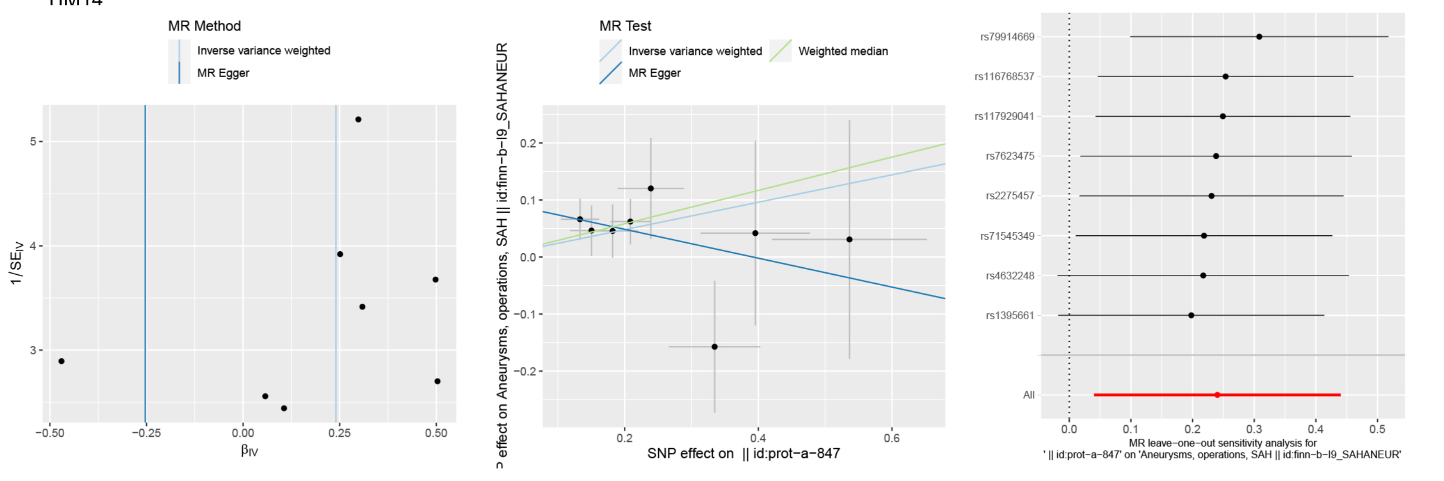

NAGS

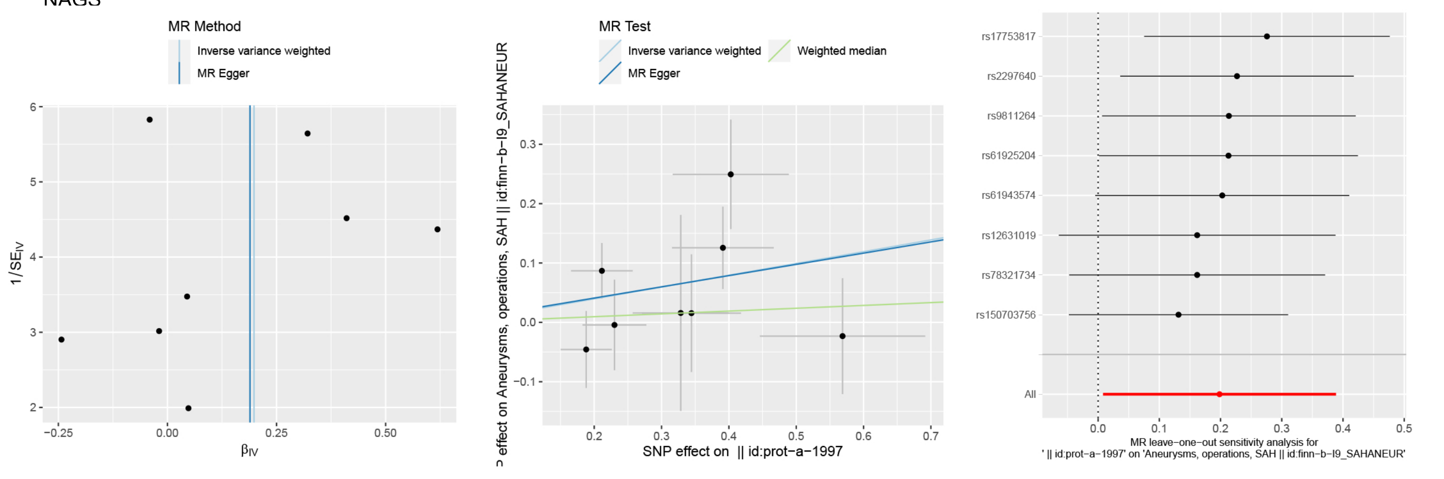

tRNA PusA

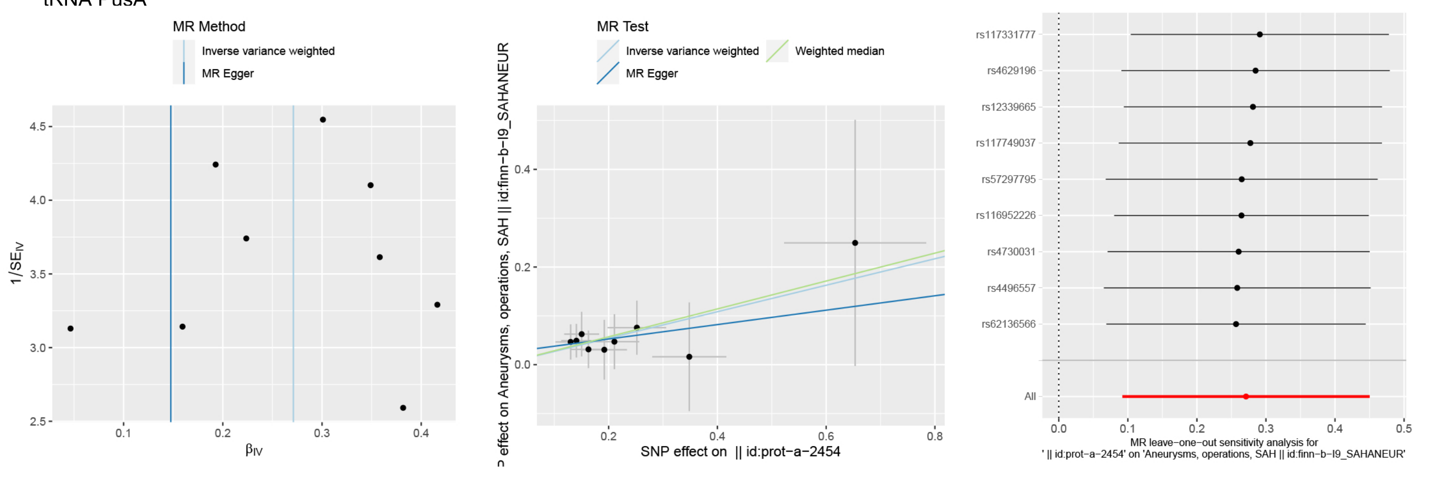

MRM3

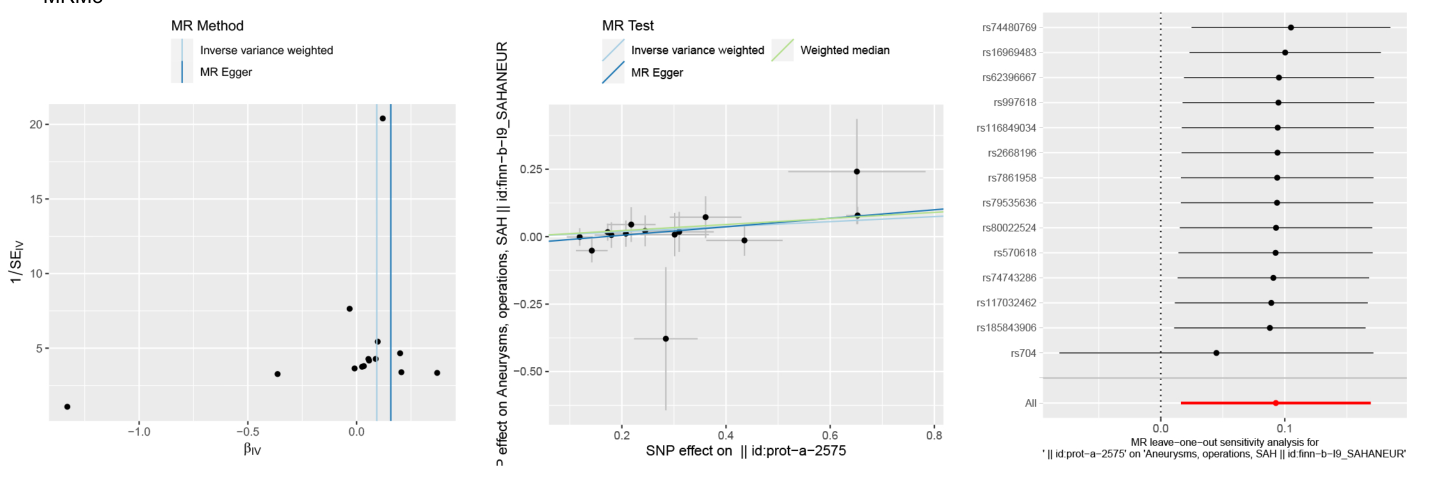

Causal effect between mitochondrial-associated proteins and uIA, including scatter plots, funnel plots and leave-one-out results

CCDC90B

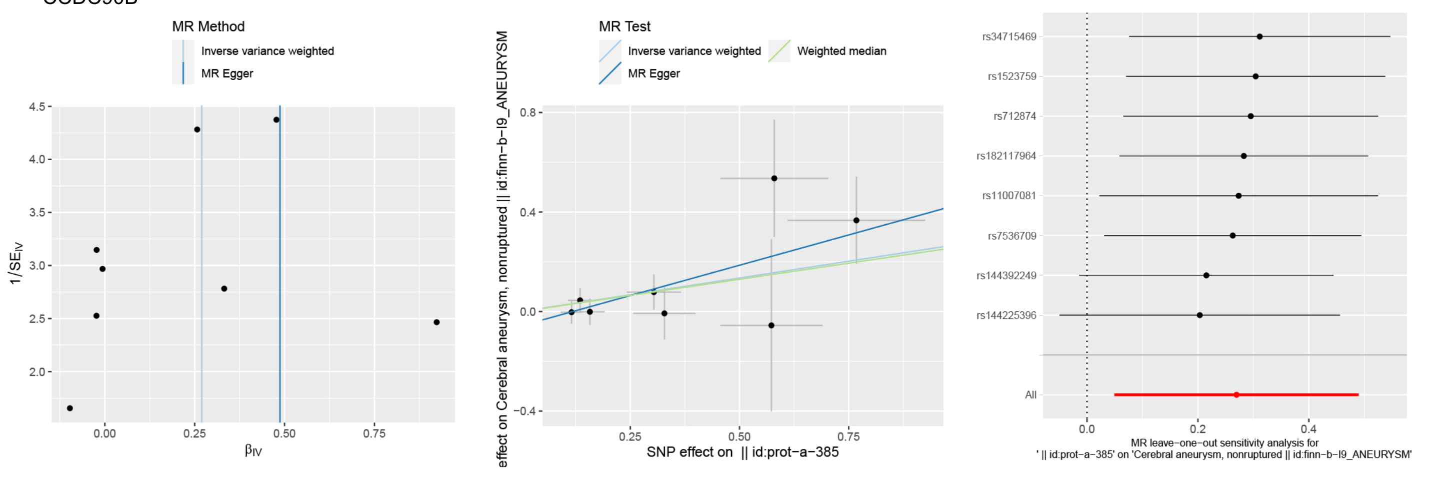

tRNA PusA

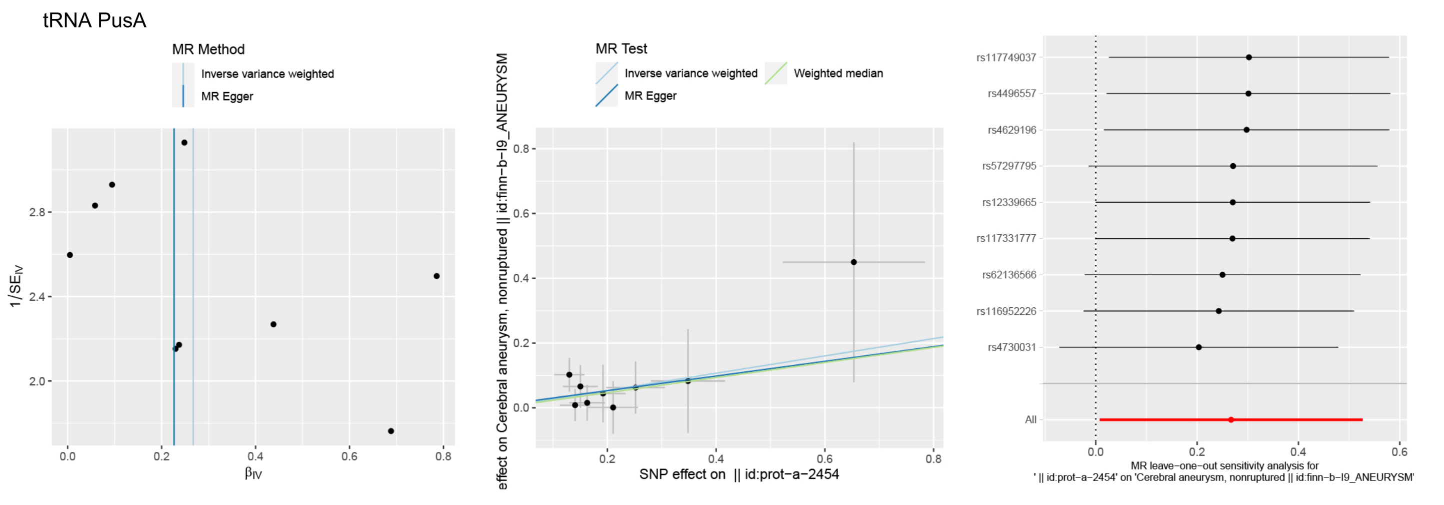

MRM3

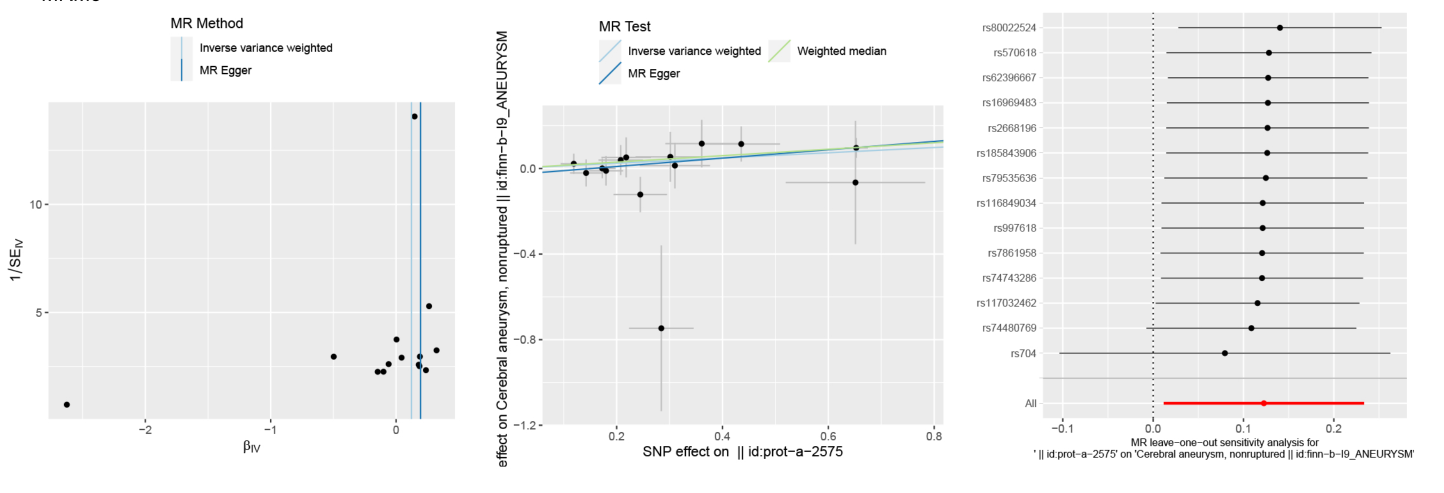

Supplement: Supplementary file 1 [file Data_Sheet_1.pdf]
